# BSSF: a fingerprint based ultrafast binding site similarity search and function analysis server

**DOI:** 10.1186/1471-2105-11-47

**Published:** 2010-01-25

**Authors:** Bing Xiong, Jie Wu, David L Burk, Mengzhu Xue, Hualiang Jiang, Jingkang Shen

**Affiliations:** 1 State Key Laboratory of Drug Research, Shanghai Institute of Materia Medica, Chinese Academy of Sciences, 555 Zuchongzhi Road, Zhangjiang Hi-Tech Park, Pudong, Shanghai, 201203, PR China; 2 Department of Applied Mathematics and Statistics, Stony Brook University, 100 Nicolls Rd, Stony Brook, NY, 11794, USA; 3 Department of Biochemistry, McGill University, 740 Dr. Penfield Avenue, Montreal, Quebec, H3A 1A4, Canada

## Abstract

**Background:**

Genome sequencing and post-genomics projects such as structural genomics are extending the frontier of the study of sequence-structure-function relationship of genes and their products. Although many sequence/structure-based methods have been devised with the aim of deciphering this delicate relationship, there still remain large gaps in this fundamental problem, which continuously drives researchers to develop novel methods to extract relevant information from sequences and structures and to infer the functions of newly identified genes by genomics technology.

**Results:**

Here we present an ultrafast method, named BSSF(Binding Site Similarity & Function), which enables researchers to conduct similarity searches in a comprehensive three-dimensional binding site database extracted from PDB structures. This method utilizes a fingerprint representation of the binding site and a validated statistical Z-score function scheme to judge the similarity between the query and database items, even if their similarities are only constrained in a sub-pocket. This fingerprint based similarity measurement was also validated on a known binding site dataset by comparing with geometric hashing, which is a standard 3D similarity method. The comparison clearly demonstrated the utility of this ultrafast method. After conducting the database searching, the hit list is further analyzed to provide basic statistical information about the occurrences of Gene Ontology terms and Enzyme Commission numbers, which may benefit researchers by helping them to design further experiments to study the query proteins.

**Conclusions:**

This ultrafast web-based system will not only help researchers interested in drug design and structural genomics to identify similar binding sites, but also assist them by providing further analysis of hit list from database searching.

## Background

Over the past decade, a significant proportion of the protein structures deposited in the Protein Data Bank (PDB) have come from the advanced high-throughput methods of various structural genomics initiatives[1,2]. Although the themes of these funded post-genomic projects differ at many levels, there is a central problem which is: how these sequences and structures are related to their functions. By increasing the structural repertoire, it is hoped that we will be able to improve our understanding of fold space and how proteins evolve new functions[3,4]. This structure-function relationship is especially essential in the drug design field where researchers try to target specific proteins involved in disease mechanisms by using structure-based drug design methods [5-7].

The number of in silico methods which can infer protein functions has grown enormously in the recent years. They can be categorized as sequence-based and structure-based[8]. Powerful BLAST-like sequence-search methods are able to transfer the function of a well-defined protein family to a protein with a high sequence similarity[9]. For lower sequence similarity instances, more subtle methods such as "profile" or "hidden Markov models" can be constructed from multiple sequence alignments and applied to find obscure patterns in the protein sequences, thus assigning a function to them [10-12]. All of these algorithms above assume that similar sequences are derived via divergent evolutions. However, this is limiting, as demonstrated by numerous studies showing that evolution in structure space is much more conserved than in sequence space[13]. This has spurred researchers to develop methods to infer functions directly from structural information. Many structure fold/domain classification databases have been compiled in efforts to build a basis for such algorithms [14-16]. Although they have assisted researchers in assigning functions to proteins with similar folds, these fold information based methods also have their drawbacks, as it is shown that sequence/structure can also dynamically change due to convergent evolution[17]. This is exemplified by many proteins involved in metabolism pathways, which although they do not have any fold similarity, all process similar metabolites. One explanation could be that they have a similar spatial arrangement of key residues in their catalytic binding sites. Such limitation further encourages researchers to develop methods based on key residues or local structural motifs.

Searching for similar local spatial patterns in structure databases is an especially challenging task, since it involves a large searching space and is usually time-consuming. Given its importance both in basic biology research and drug design, several algorithms have been devised to tackle this difficult problem of finding similar functional sites in structures [18-24]. Among these local spatial similarity detection methods, some rely on the curated local structure patterns, like in TESS and SPASM systems, and identify the similar local structures by comparing these curated structure templates with the query. Others are more flexible and able to take many structures into account, then build the structure patterns during the process and detect the similar local structures on the fly as exemplified by Hamelryck's multidimensional index tree method[25]. These methods not only give hints about structure evolution and protein functions, but also play a significant roles in predicting drug side-effects caused by ligand cross binding to similar surface patches on various target structures. At the fundamental level, the elementary searching algorithms used in these methods are usually based on geometric hashing and graph clique detection. Due to the significant computational expenses of these algorithms, they typically rely on previously calculated data sets and it would be nontrivial to apply them within the whole structure space represented by the PDB. Users can only visualize and analyze results stored in a pre-compiled database. While as a consequence of structural genomics research, there is a continuously increasing demand for performing binding site similarity searches with user input structures in order to find possible functions for these binding sites as well as for the proteins in a large scale and fast way.

Here we present a new ultrafast binding site similarity search method along with a simple analysis of occurrences of Gene Ontology (GO) term and Enzyme Commission (EC) numbers in hit list[26]. Our method was inspired by the fingerprint and pharmacophore concepts commonly used in chemoinformatics[27]. We first systematically extracted possible binding sites from protein structures, mapped pharmacophore properties to the binding sites and calculated fingerprints for later database searches. To enable users to identify subtle similarity between binding sites, a panel of fingerprint measurements was tested to find the optimal solution. Further, a statistics-based score method was developed to evaluate hits with the aim of eliminating false positives. This novel binding site similarity search method should enable researchers in the structural biology field to examine in detail for possible binding sites in an ultrafast large scale way. Also it will benefit researchers in the field of drug development by allowing them to predict and investigate possible side effects due to ligand cross binding events.

## Methods

### Extract possible binding sites from PDB database

First, a total of 41449 protein structures were gathered from the RCSB PDB (up to 2008.1)[28]. Then, a geometry-based protein binding site detection method, named PASS (Putative Active Sites with Spheres)[29], was adopted to extract possible binding sites from every polypeptide chain in the database of protein structures. This tool is able to characterize concave regions of a protein surface and to identify positions likely to represent binding sites based upon the size, shape, and burial extent of these volumes[29]. The output spherical probes of PASS were processed with an in-house program based on a minimum spanning tree algorithm to split the probes around the protein surface into clusters at least 5 Å apart. This resulted in 201,233 probe clusters, which were further filtered so that only probe clusters containing 30 to 200 probes were considered as binding sites. These feasible binding sites were used to extract protein residues within 6 Å of these probes. These binding site probes along with the identified binding site residues were stored into the database for later visualization and fingerprint calculation. Two other datasets which compiled with known small molecular binding sites were also constructed for a better evaluation of our method. First, the HETATM residues in PDB database were filtered such that only the ones which have molecular weights between 100 and 800 were retained. Also, if one residue shows up for more than 10 times in PDB database, then it will be regarded as a common molecule and discarded. Finally, 13227 PDB structures were subjected as a database for later geometric hashing and fingerprint calculation. In geometric hashing calculation, only the backbone CA atoms and centroids of functional fragments of side chains of binding site were retained. (See the additional file 1, Table S1 for definitions of fragment types). We named this dataset GH Validation Dataset. The other dataset is FP Validation Dataset, which use the known binding sites of these 13227 PDB structures to calculate the fingerprint with the method describe below.

### Fingerprint calculation

**Figure 1:**
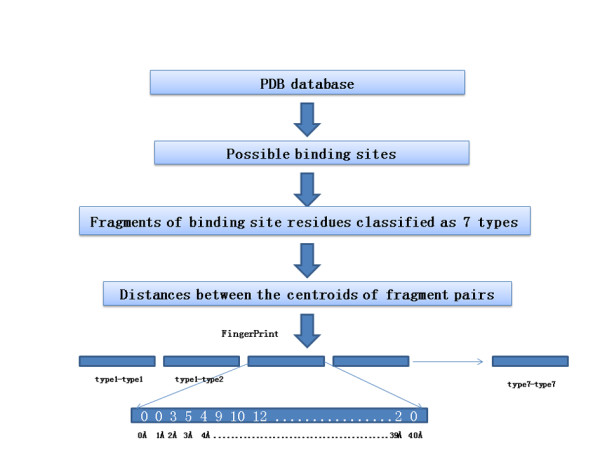
**Workflow to compute a fingerprint from a binding site structure**. The atoms of the binding site residues are divided into 7 fragment types according to their properties. Distances between the centroids of each pair of fragments were then calculated and the distances are recorded and binned into the corresponding locations in the fingerprint (FP).

A fingerprint, commonly utilized in chemical similarity research, is a string representation of molecular structure and properties. Normally, fingerprints take account for atomic distance or connectivity patterns. And a comparison of fingerprints can provide an inexpensive way to obtain similarity comparisons between query and reference structures. Given that the aim of the present work is to develop a fast method for binding site similarity searches, we borrowed the concept of fingerprint to characterize binding site in proteins. Generally, each amino acid was split into several fragments (see additional file 1, Table S2 for details), and these fragments were classified according to 7 physicochemical types: hydrogen bond donor/acceptor(type 1), hydrogen bond acceptor(type 2), hydrogen bond donor(type 3), aromatic(type 4), lipophilic(type 5), positive charged(type 6), negative charged(type 7). The fragments of the binding site residues were mapped into these pharmacophore-like types and the centroid of each fragment was calculated. The distances between each fragment-fragment centroid were binned into 0-40 Å categories with a 1 Å stepsize (the procedure is illustrated in Figure 1).

### Fingerprint scoring function

The heart of any similarity comparison system is the measurement method for quantifying the degree of resemblance between two objects. Several binary and quantitative fingerprint scoring functions were assessed including Tanimoto Distance, Euclidean Distance, Canberra Distance and Angular Separation. The Canberra Distance (Equation 1) was identified as giving the best performance measurement:(1)

which denotes the dissimilarity score between fingerprint i and j, both of which have n bins.

Obviously the number of fragments in a binding site will affect the score of the fingerprint similarity function (the greater the number of fragments, the larger the count in the fingerprint). The relationship between fragment number and the dissimilarity score should be investigated. In the present work, we first randomly selected 3000 binding sites from our database and then calculated the dissimilarity scores between these fingerprints. Let the fragment numbers of two binding sites i and j be denoted as *Num*_*i *_and *Num*_*j*_. Considering the algorithm of the scoring method, we calculated a new *Fix_Num*_*ij *_for each two binding sites:(2)

Scores (*FP_Score*) below 100 (which indicate similar binding sites not representing random comparison pairs) were eliminated and remaining scores were stored into bins of *Fix_Num*_*ij *_(window length 0.002), where for each bin the averages and standard deviations (SD) were computed. This is similar to the procedure of regress 3 in Pearson's FASTA sequence alignment scoring function[30]. Then least square fitting was carried out for the histogram data of the averaged scores and SD against the fixed fragment number Fix_Num_*ij*_. The fitted *Mean*_*fit *_and *SD*_*fit *_can be calculated for each *Fix_Num*_*ij *_using the parameters obtained from the fitting. Now the Z-score for every pair *ij *can be defined as:(3)

Thus, a comparable Z-score is available for selecting similar binding sites from the fingerprint database.

## Results

### Analysis of possible binding sites

The goal of structural biology is to investigate and understand protein functions through three dimensional structures. Proteins execute their functions via binding to other cellular components such as ligands. The contact points located on the protein surface are commonly known as binding sites. Although it still remains a challenge to identify binding sites solely from a three-dimensional structure, several computational methods have been developed to detect such spatial motifs. One of them, PASS[29], is a binding site detection program based on an analysis of the geometric features of the protein surface. Due to the great difficulty of identifying biologically meaningful binding sites, we decided to gather all possible binding sites for the analysis. At first, a total of 41449 structures were retrieved from the PDB[28] database and filtered to remove all non-standard amino acids. Next, each chain containing more than 100 amino acids was saved as a file to be used in binding site detection using the PASS program, which resulted in 201233 possible binding sites, or roughly two binding sites per polypeptide chain. A detailed analysis of these possible binding sites shows that the average size of a binding site is 30 amino acids (summarized in additional file 1, table S3). Mapping the binding site residues to pharmacophore fragments identifies about 88 fragments per binding site. By analyzing the pharmacophore type distribution in the binding sites, it is clearly shown that both hydrogen bond donor/acceptor and aromatic fragments are enriched in the binding sites compared to the whole proteins, while not the lipophilic pharmacophores.

**Figure 2:**
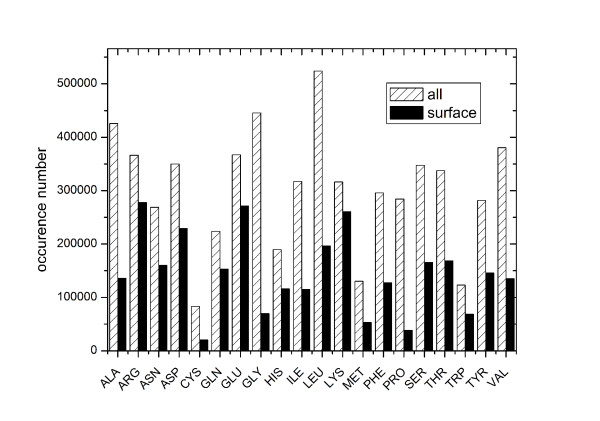
**Binding site residues distribution**.

To further examine the amino acid distributions in binding sites, we also investigated the amino acid occurrence both for all the residues in binding sites (within 6 Å of the PASS probes) and only solvent accessible residues. As shown in Figure 2, the amino acid distribution pattern of whole binding sites is consistent with the fragment types found in the binding site, while the distribution of solvent-accessible residues are quite different. In binding sites, the positively/negatively charged residues (ARG, LYS; ASP, GLU) occur more frequently, while the amino acids CYS and PRO are rarely found. This is consistent with an analysis of the catalytic residues in enzyme binding sites. Although in enzymes, histidine is also a critical player in catalysis[31].

### Similarity scoring system

Due to the difficulty of obtaining a gold standard benchmark of similar binding sites in a meaningful scale, we use simulated datasets described below as a control to identify the most appropriate measurement for those fingerprints containing modest similarity. We first grouped the binding sites in the database according to their fragment numbers, denoted as *Group*_*n *_(n is the fragment number in the binding site). For each *Group*_5×*i*_(*i *= 6, 7, 8, ..., 40), we selected 10 binding sites randomly. Two synthesized binding site datasets were created with the following strategies.

#### Dataset I

For each binding site *S *(with fragment number *Num*_*s*_), we created 100 new structures denoted as *(k = 1,2,3, ..., 100)*. In each , the previous structure of S was kept and  random points were added with the follow procedure:

• Fragment types are selected randomly from 1 to 7;

• Two farthest points in binding site are located, and then two spheres of 8 Å radius are created centred by these two points;

• Randomly set point coordinates inner the spheres until certain number of points have been got, every point should be separated by at least 3.5 Å.

#### Dataset II

This dataset is derived from Dataset I. The atom types in  are re-defined randomly while the coordinates of binding site structures are retained. We consider this dataset as a random dataset.

**Figure 3:**
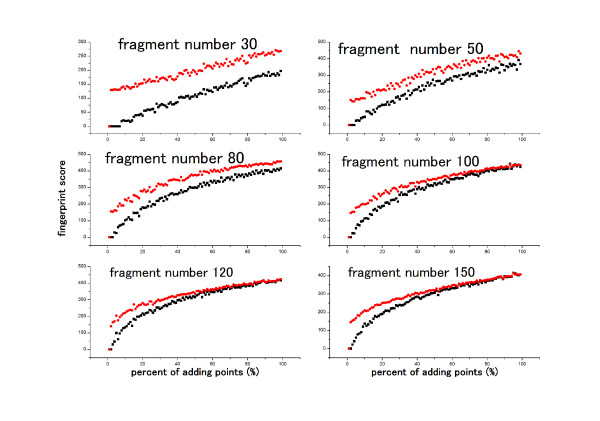
**Raw fingerprint scores for two simulated binding sites data sets**. Here only six groups are shown. The black are scores of Dataset I. The red lines are scores of random Dataset II.

Several binary and numerical fingerprint similarity measurement methods were assessed using these two datasets with the aim of finding one that could separate the similar binding sites from the random ones. The influence of bin step size (0.1 Å, 0.5 Å, 1 Å, 1.5 Å, 2 Å) in the fingerprint calculation was checked, and it showed that a 1 Å bin size was detailed enough to give a good description of binding site shape. After investigating the similarity measurement methods, the Canberra Distance (see Method Section) was found to be the most appropriate scoring function in our case. As shown in Figure 3, this similarity measurement method is capable of separating partially similar binding sites from random binding sites when they have similar sizes, especially when the fragment number in the original binding site is less than 100. Even when the binding sites are large, this scoring function still worked if the fragment numbers added do not exceed 50% of the original fragment number.

Although the Canberra Distance scoring method outperforms other fingerprint scoring methods in this simulation, it was clearly not feasible to separate similar binding sites from random ones if the binding sites were in different groups. This spurred us to devise a Z-score function to correct the similarity score calculated for different binding sites.

**Figure 4:**
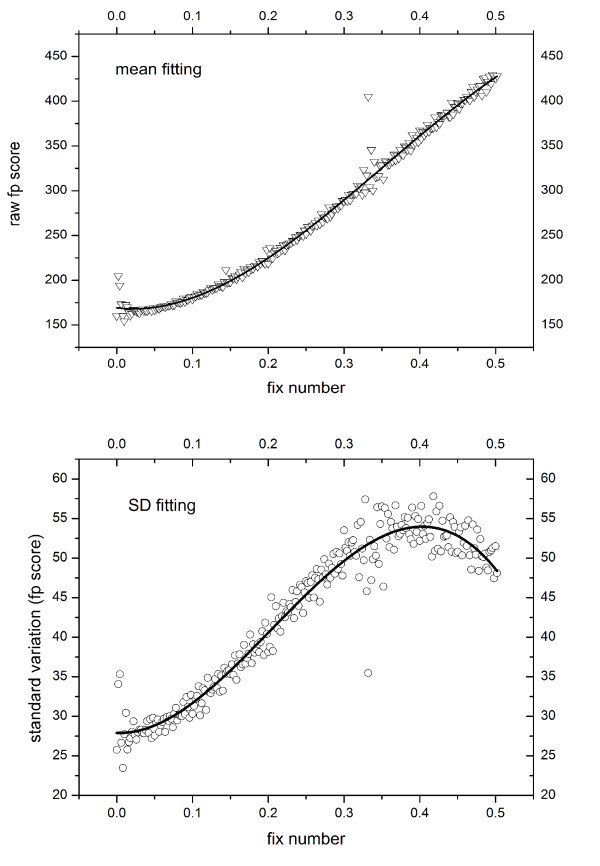
**Least square fitting of the mean and standard deviation of the raw fingerprint scores**.

For the sake of generalization, we randomly selected 3000 binding sites from our binding site fingerprint database and calculated similarities for each pair (9,000,000 pairs). After removing obviously similar pairs *(FP_Score < 100.0*), the histograms of the mean and standard deviation versus the *Fix_Num*_*ij *_were calculated with the procedure of regress 3 in Pearson sequence alignment score function[30]. Only the number of score data points in the histogram which are larger than 5000 were considered reliable and used in later fitting. As depicted in Figure 4, the smooth line for the mean value is obtained from the cubic polynomial fitting with an R-square value of 0.991. The standard deviation fitting is slightly worse with an R-square value of 0.953.

With the parameters obtained above, the mean and standard deviation of the score at a given *Fix_Num*_*ij *_can be defined as the following:(4)(5)

The Z-score can then be computed with Equation 3.

To evaluate the Z-score performance, Dataset I and II were used again to recalculate the Z-score for them. It was found that the Z-score for the similarity of the random binding sites were around 2.5, except for the case of very small binding sites. This should allow users to better judge the results from database search.

**Figure 5:**
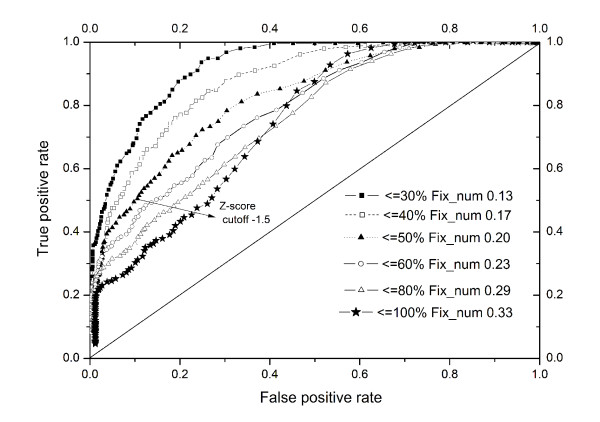
**ROC curves calculated from the Dataset I and II. to assess the boundaries of Z score cutoff and fix number**.

In general, throughout the analysis of the simulated datasets, the more points added to the original binding sites, the more difficult it is to detect the subtle similarity between them. Also, the Z-score scheme needs a cut-off value to indicate cases of reliable similarity when searching the binding site database. To accomplish this critical assessment for the boundary of the Z-score function, the Receiver Operating Characteristic (ROC) curve method was adopted[32]. In order to calculate the ROC curves, we randomly chose 1000 binding sites from the simulated Dataset I and labelled these binding sites as the true similar group. Another 1000 binding sites randomly chosen from Dataset II were labelled as the false group. We then varied the Z-score cut-off (from -5 to 5 with a step size of 0.1) to calculate true positive (TP), false positive (FP), false negative (FN) and true negative (TN) values. After that, the true positive rate (TPR) and false positive rate (FPR) were calculated following equation 7,8 and plotted as a ROC curve (Figure 5, stars).(6)(7)

The TPR represented the sensitivity while the FPR is the 1-specificity.

As demonstrated in Figure 5, it is not surprising that the classification ability is limited in some cases. Although at lower Z-score cut-offs (from -5 to -2) the TPR is dominant, the similarity detection ability falls off as long as the Z-score cut-off rose up. To take account of the effects of added points on Z-score performance, we also randomly chose 1000 binding sites from Dataset I and converted binding sites with an added point percentage larger than 80% to the false group. Combined with another 1000 randomly chosen binding sites from Dataset II, the ROC curve was calculated again and plotted as the 80% line in Figure 5. Similarly, we calculated 60%, 50%, 40% and 30% lines and plotted them in Figure 5. Clearly and intuitively, the power of the classifier increases as the more subtly similar binding sites are assigned to the false group. From the ROC curve, it was illustrated that even when we added 50% random fragments to the binding sites, the Z-score scheme was still able to give encouraging results. With a Z-score cut-off of -1.5, the true positive rate is about 50% and the false positive rate is only 10%.

### Assessment with known ligand binding sites

To validate our fingerprint scoring strategy, a geometric hashing based similarity measurement was implemented for a comparison. Geometric hashing is a well known sequence-independent 3D similarity searching method, which has been adopted as a basis by web servers like SitesBase[33]. 450 PDB entries were randomly selected from known binding site dataset. Then they were searched by both geometric hashing and our fingerprint scoring method, against GH and FP Validation Dataset respectively. Totally 431 PDB binding sites were successfully processed by both methods.(All the validation results and softwares can be accessed at our web site http://202.127.30.184:8080/bssf/validate_exp.jsp and http://202.127.30.184:8080/JChem/li/indexGH.jsp).

The output from geometric hashing method was categorized into three levels of similarities according to the percentages of matched points with the query (>1/3,>1/2 and > 2/3). In this test, 11121, 4309, 1568 pairs of similar PDB entries were found at 1/3, 1/2 and 2/3 levels respectively.

We also defined a manner to classify the results from fingerprint based method. For each query, first, we counted PDB entries which were found as similar by geometric hashing method in certain level, this number was set as N_sim_. Then the searching result of fingerprint method was sorted by the Z-scores from lowest to highest. After we get the numbers, the sorted list of fingerprint search result will be truncated so that only the first N_sim _× N_fold _(N_fold _= 1,2,3,4,5) entries will be kept. Then the truncated list was investigated by counting the PDB entries(N_found_) which were considered as similar in geometric hashing measurements. Finally, the number N_found_/N_sim _was used as a success ratio to assess the fingerprint Z-score strategy.

**Figure 6:**
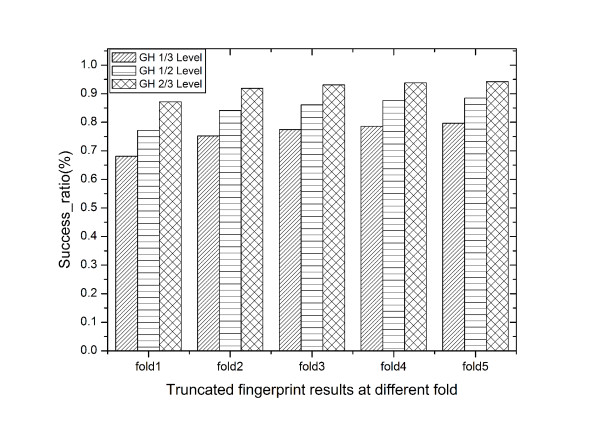
**Comparison of the geometric hashing method with the fingerprint Z-score method**.

As demonstrated in Figure 6, even when truncated at 1 fold, the fingerprint result is still promising. If we use the geometric hashing level 2/3 as the positive data, at N_fold _= 1, the fingerprint method can detect about 87% PDB entries from the geometric hashing similar list. At N_fold _= 2, it can detect about 92% PDB entries. If lower similarity level in geometric hashing method was selected as positive data set, the PDB entries detected by fingerprint method also diverge gradually. Through Figure 6, one can found that the results from N_fold _= 2 are almost the same as the five fold, which clearly shows the nature of rapid convergence of this fingerprint method. We also checked the average Z-scores among the truncated list. For the truncated list from N_fold _= 1 fold, they are -4.3, -5.2 and -6.0 at the 1/3, 1/2, 2/3 level respectively. While for the fold two truncated list, the average Z-score values are slightly up to -3.4, -4.1 and -4.8.

Taking a close look at the results from geometric hashing and fingerprint methods, it is clear that they can complement each other. For example, According to the PDB annotation, the entry 3H4A is E.coli 6-Hydroxymethyl-7,8-dihydropterin pyrophosphokinase (HPPK) complexed with AMPCPP[34]. From the geometric hashing searching, total 11 PDB entries were found having similarities better than 1/3 level (Matched points more than 1/3 of query binding site). Blast searching the sequence of 3H4A against these 11 PDB also shows that all of them have Blast E-value which are lower than 1E-50. This demonstrated our geometric hashing procedure is capable to find the similar binding sites. Interestingly, after investigating the fold two truncated list from fingerprint method, we found this list not only covers the 11 entries found above, but also includes some other entries, like 1CBK and 2BMB (which have low blast E-values 4E-47 and 1E-17 with 3H4A), 1C85, 1C86 and 1C88 (all of which are protein-tyrosine phosphatase 1B)[28]. All of these extra entries have low fingerprint raw scores and Z-scores but not show up in either geometric hashing.

**Figure 7:**
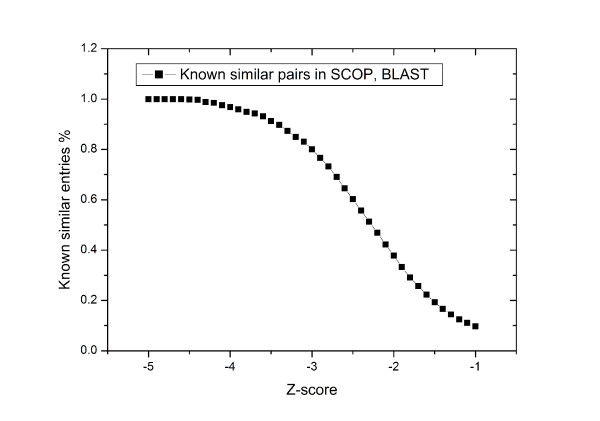
**Validation the fingerprint Z-score method with SCOP and Blast Dataset**.

We also incorporate SCOP classification and blast E-values to check the sensitivity and robustness of our fingerprint Z-score method, since both of topology and sequence similarities can partly imply a possibility of binding sites similarity. PDB entries from known binding site data set were grouped based on sequence identities and SCOP family terms. Then for each of the 431 PDB entries, we checked the results from our method with these reference groups for overlaps. As shown in Figure 7, at low Z-score value, the similar pairs found indeed have high sequence similarity. This further demonstrated the ability of our fingerprint scoring system in detecting the obviously similar cases. At Z-score cutoff -3.0, about 80% predicted similar pairs are either contained in the same SCOP family or have the sequence blast E-value lower than 1E-10. However, the other 20% cases, which also have strong similarity Z-scores, can not be simply regarded as false positive, because two proteins may have similar shape of binding sites but don't have much similarity at the overall level. For example, in this study, from the result list of PDB entry 1DJY, 1PCM has a Z-score -3.15. But these two proteins do not show any relation on either sequence similarity (E-value 1E-10) or SCOP classification. Through detailed examination of their active sites, it was found that both of them contain a metal atom and phosphate sugar-like ligands, which implies that their binding sites have certain similarity(Please refer the additional file 1, Figure S1).

### Comparison with other servers/softwares

Finding similar binding sites is an important issue in the field of computational structural biology. It is not only useful in rational drug design, but also can provide information on protein functions. To evaluate the performance of our fingerprint scoring method, we also compared it with several web servers and softwares. The PocketMatch dataset was used in this comparison[35]. As listed in Table 1, the fingerprint Z-score method is effective in all the cases except the 1ZID_ZID-2CIG_1DG pair, for which PocketMatch has a low PMC_min _Score. Also, the comparison shows that in several cases, the SuMo and geometric hashing method used in SitesBase fail to identify the similarity of the pairs. while the fingerprint Z-score method is able to find the relations. Clearly, the Z-score scheme is necessary in fingerprint method. For example, the 1GJC_130 and 1V2Q_ANH have a raw fingerprint score 194.09, which is high enough to recognize as similar. But the Z-score method normalizes this raw score and gives the -4.00, a value strongly suggested the similarity relationship between two binding sites.

**Table 1:** Case studies by comparison with other servers/softwares.

PDB1	PDB2	ProFunc	SuMo	SitesBase		PocketMatch		PyMol	BSSF	
		**Score**	**Score**	**Score**	**PI**	**PMScore_Min_**	**PMScore**	**rmsd**	**FP_Score**	**Z_score**

**Cases from same SCOP families**										

1DHJ_MTX	4DFR_MTX	112	100	83.33	0	85.25	85.25	0.231	61.91	-3.87

1A4G_ZMR	1NSC_SIA	112	76	93.67	0	99.91	88.39	0.141	85.58	-2.90

1SDU_MK1	1SDT_MK1	X	NA	100	0	98.93	88.39	0.298	40.34	-5.00

1B42_SAH	2VP3_SAH	241	91	76.92	0	99.4	89.29	0.088	77.96	-3.20

1GJC_130	1V2Q_ANH	229	89	29.38	3E-28	93.65	50.17	0.294	194.09	-4.00

1GJC_130	2AYW_ONO	217	40	NA	NA	56.9	52.29	1.186	394.22	-1.42

1GJC_130	1O3P_655	X	NA	54.80	0	100.0	88.01	0.113	87.31	-2.95

1ADD_1DA	2ADA_HPR	150	45	93.06	0	94.72	83.59	0.149	77.22	-3.21

1KV5_PGA	2JGQ_PO4	104.58	X	NA	NA	80.48	28.40	3.527	83.63	-2.98

1BZC_TP1	1GFY_12P	174	27	60	2.4E-40	96.87	75.41	0.236	86.52	-2.94

1DJX_13P	1DJY_12P	X	58	86.79	2.5E-38	100	69.05	0.160	61.35	-4.16

1AJ6_NOV	1EI1_ANP	102	X	55.55	7.6E009	91.53	21.16	3.019	214.00	-1.78

**Cases from different SCOP families**										

1ECM_TSA	4CSM_TSA	X	X	54.65	8.6E-27	74.22	55.56	0.640	84.86	-3.02

1M6Z_HEC	1LGA_HEM	X	X	X	X	67.58	63.85	5.875	149.16	-0.66

1ZID_ZID	2CIG_1DG	X	X	X	X	58.94	56.01	5.691	176.40	0.27

1V07_HEM	1HB1_HEM	X	X	46.81	2.6E-16	68.94	61.42	0.690	144.04	-0.92

### Web server

A supporting website http://202.127.30.184:8080/bssf/ was constructed for user-defined calculations and predictions. The web site was built with Java JSP technology and the analysis and prediction procedure consist of four steps: 1) The user supplies a newly determined crystal structure or a PDB entry ID to conduct the analysis. Following submission of the data, the binding site detection and fingerprint calculations are initialized. 2) The user can visualize the binding sites in the crystal structure using the web embedded Jmol[36] program. 3) The user can select the binding site to perform a database search with the Z-score scheme. After finishing this fast database search (normally about 1 minute), the result will appear in a table on the web page for inspection. The user can download the result for later analysis or further query the NCBI with blast program, checking the PDB entry in PDB database. Also, the user can take further steps to analyze the hit list to check the occurrences of some basic information such as GO terms and EC numbers. These metric may benefit researchers to design new experiments to study the query protein at hand.

## Discussion

A major goal of structural biology is to understand cellular functions in the context of the atomic details of molecules. With increasing deposits of three-dimensional structures in the RCSB Protein Data Bank through structural genomics initiatives, there is a pressing requirement for experimental or computational methods to correlate functions to these structures. This sequence-structure-function relationship underlies the numerous investigations aimed at dissecting the biological properties of proteins.

In the present work, we describe a fast method for detecting similar binding sites in protein structures in the whole PDB database. This may shed light on protein function and possible drug side-effects due to ligand cross binding to similar sites. Our method is developed for 3D local structure similarity detection and complements sequence-based or fold-based methods. It can uncover similarities in small spatial surface regions on protein structures and provide additional evidence for inferring protein functions[37]. In contrast to many 3D motif similarity searching methods, we use a fingerprint approach to represent the binding sites. The fingerprint concept is heavily implemented in the chemoinformatics field for small molecule database searching and has been proved to be fast and useful in ligand similarity research. We have extended it to label binding sites in macromolecules. To simplify the large number of atoms in binding sites and to implicitly add flexibility for fingerprint representation, every residue in each binding site was fragmented into subgroups and mapped to 7 properties similar to the pharmacophore concept. This fingerprint representation not only eliminated the sequence order dependence usually encountered in sequence/structure similarity measurements, but also enabled an ultrafast method of searching a comprehensive binding site database. The time consumed to perform a single query with our binding site database (188959 entries after the binding sites smaller than 30 points or larger than 200 points being filtered) is approximately 1 minute on an Intel XEON 2.8 G processor. This gives researchers tremendous opportunities to conduct large scale comparison studies to elucidate functions for any possible binding site.

The method described here differs substantially with structure-template based methods. In these methods, local structure-template are curated from the protein structures and usually only contains very few residues, exemplified in TESS system three residues catalytic traid "O-HIS-O"[24]. Although the method presented here also needs to extract the binding sites from PDB structures in advance, the binding sites are not limited to a fixed number of residues. Based on the fingerprint concept, variable binding sites can be represented and compared without any difficulty. Such circumstances would be very time-consuming with the graph clique algorithm or geometric hashing algorithm based methods [22,23]. Recently, Xie and Bourne, based on the weighted graph maximum clique detection algorithm, devised a method SOIPPA, which can find the similar functional sites through sequence order-independent profile-profile alignment[38]. Through implemented several heuristic rules, authors accelerate the functional matching phase and simultaneously found and aligned the similar binding sites. But due to that the intrinsic algorithm is based on the graph maximum clique detection, the running time still beyond the routine database search for the whole PDB database with all the possible binding sites, especially in the situation of fast growing of the structures out from the structural genomics project.

In comparison to the similar very fast method pvSOAR[18], which also extracts possible binding sites with an automatic alpha shape method, our method is sequence-order independent and does not take into account the local sequence similarity between the two binding sites. This represents a more natural way to describe the shape and properties of a binding site, especially where two binding sites only share sub-pocket similarity. WebFeature is another ultrafast binding site similarity comparison and functional annotation system[39]. It uses the calculated biophysical properties of binding sites to represent the binding site and utilizes a machine learning method to train the system and predict the function for the query. Compared to their approaches, our method is merely based on the original PDB structure data and does not go through the training phase. Also the WebFeature method is based on the already determined functional motif stored in PROSITE database, then may not cover all the possible binding sites represented in the PDB structures.

The binding site database in our method could be further improved to expand coverage and accuracy. In the current implementation, only binding sites that involve single polypeptide chains are taken into account. We do so mainly because it is very difficult to separate true multi-chain complexes from artefacts due to crystal packing interactions in a unit cell. Nevertheless, including such binding sites in our database will expand its coverage and enhance function inference. Another drawback of our method is that the PASS predicted binding sites may not be the true binding sites on the protein surface. This may increase the false positive rate and reduce prediction power. Although there exists such computational methods to identify the true binding sites, this shows to be a very difficult task due to the limitation of our knowledge of possible protein-ligand interactions which exist in nature.

A major challenge in analyzing local spatial patterns is how to assess the significance of the detected similarity. Due to the difficulty to obtain the gold standard of the binding site data sets, we decided to use the simulated binding sites as the representation for later statistical judgement. To overcome the limitations of the original fingerprint Canberra Distance score function, we devised a Z-score scheme and investigated its boundary in detail by gradually changing two variables, namely Z-score cut-off number and Fix number. It was found from the ROC curve that the performance is promising even at a Z-score cut-off value of -1.5 and with less than 50% added random points. This validation strengthens the utility of our method and provides guidelines for later database searches. As demonstrated by the ROC curve, our method has the capability to detect sub-pocket similarity. It is very important in drug design, to detect such weak similarity, since a ligand may only interact with a few key residues in a binding site to execute its biological role. This will help researchers to identify possible targets similar to known drug targets and to predict side-effects for certain drugs.

In future, one important further extension of our method is to combine it with other sequence-based or structure-based function inference methods to enhance accuracy in assigning functions. Recently Brylinski and Skolnick provided a method named FINDSITE[40], which can locate the binding sites in protein structure through a threading alignment of distant homologies. Their method can successfully identify 70.9% binding sites in the top five predicted binding sites. Although the prediction power dropped down when the sequence identities of homologies are below 35%, combinations with the fold information or sequence information could improve the prediction accuracy[41]. Like in the comprehensive protein functional annotation database ProKnow[42], Pal etc. integrate information about the query protein and then weighted the information in a Bayes framework. Their investigation clearly demonstrated that the multiple sources of information will enhance the prediction power. Given the ability of our ultrafast binding site similarity method, it could be assembled with others sequence and structure similarity measurement and improves the prediction for the binding site functions.

## Conclusions

It is well recognized that sequence and structural fold are dynamically changing under evolutionary pressure over long time scale. These diverging and converging evolutionary phenomena produces a challenging problem of how to infer the functions of newly discovered genes from their sequences and structures. Many sequence-based and structure-based methods have been developed to correlate functions to sequences and structures and to extend our ability to understand the fundamental relationship between sequence, structure and function. Although some cases can be easily solved, some more difficult cases often just contain very weak sequence and structure similarity with proteins with known functions in curated databases. As a consequence, there is an ongoing need of novel methods to broaden our capability to predict function in this post-genomics era. Here we presented a novel and fast binding site similarity detection and function inference system. By utilizing fingerprint representations of binding sites, we are able to conduct an economical similarity measurement. Furthermore, for the accurate detection of similar binding sites, especially ones where there is only weak or sub-pocket similarity, a statistical validated Z-score scheme was devised to improve sensitivity. This system could be used in the drug design field to identify promising targets for drugs by using the binding site of its known target as a query. It could also benefit researchers in the field of structural biology field by allowing them to find similar structures at binding site level.

## Authors' contributions

BX and JS performed the design of the study. BX and JW programmed the software and constructed web site. JW and MX performed the PDB structures processed and extracted binding sites. DLB, HJ and JS performed analysis, test the web site and conducted the case study. BX, JW and DLB wrote the draft. Finally, all the authors read and approved the final manuscript.

## Supplementary Material

Additional file 1 **Supporting material**. It includes the fragment types in geometric hashing method and pharmacophore fingerprint. Also it contains the properties of binding sites. Click here for file

## References

[B1] Warr WA Future structural genomics initiatives: an interview with Helen Berman, director of the Protein Data Bank Journal of Computer-Aided Molecular Design 2008 22 10 707 710 10.1007/s10822-008-9234-3 18781281

[B2] Kouranov A Xie L De la Cruz J Chen L Westbrook J Bourne PE Berman HM The RCSB PDB information portal for structural genomics Nucleic Acids Research 2006 34 D302 D305 10.1093/nar/gkj120 16381872 PMC1347482

[B3] Godzik A Jambon M Friedberg I Computational protein function prediction: Are we making progress? Cellular and Molecular Life Sciences 2007 64 19-20 2505 2511 10.1007/s00018-007-7211-y 17611711 PMC11136138

[B4] Redfern OC Dessailly B Orengo CA Exploring the structure and function paradigm Current Opinion in Structural Biology 2008 18 3 394 402 10.1016/j.sbi.2008.05.007 18554899 PMC2561214

[B5] Buchanan SG Structural genomics: Bridging functional genomics and structure-based drug design Current Opinion in Drug Discovery & Development 2002 5 3 367 381 12058612

[B6] Lundstrom K Structural genomics: the ultimate approach for rational drug design Mol Biotechnol 2006 34 2 205 212 10.1385/MB:34:2:205 17172666

[B7] Veber DF Drake FH Gowen M The new partnership of genomics and chemistry for accelerated drug development Current Opinion in Chemical Biology 1997 1 2 151 156 10.1016/S1367-5931(97)80003-8 9667859

[B8] Lee D Redfern O Orengo C Predicting protein function from sequence and structure Nature Reviews Molecular Cell Biology 2007 8 12 995 1005 10.1038/nrm2281 18037900

[B9] Altschul SF Madden TL Schaffer AA Zhang JH Zhang Z Miller W Lipman DJ Gapped BLAST and PSI-BLAST: a new generation of protein database search programs Nucleic Acids Research 1997 25 17 3389 3402 10.1093/nar/25.17.3389 9254694 PMC146917

[B10] Bateman A Birney E Cerruti L Durbin R Etwiller L Eddy SR Griffiths-Jones S Howe KL Marshall M Sonnhammer ELL The Pfam Protein Families Database Nucleic Acids Research 2002 30 1 276 280 10.1093/nar/30.1.276 11752314 PMC99071

[B11] Engelhardt BE Jordan MI Muratore KE Brenner SE Protein molecular function prediction by Bayesian phylogenomics Plos Computational Biology 2005 1 5 432 445 10.1371/journal.pcbi.0010045 PMC1246806 16217548

[B12] Soding J Biegert A Lupas AN The HHpred interactive server for protein homology detection and structure prediction Nucleic Acids Research 2005 33 W244 W248 10.1093/nar/gki408 15980461 PMC1160169

[B13] Chothia C Lesk AM The Relation between the Divergence of Sequence and Structure in Proteins Embo Journal 1986 5 4 823 826 3709526 10.1002/j.1460-2075.1986.tb04288.x PMC1166865

[B14] Holm L Sander C Mapping the protein universe Science 1996 273 5275 595 602 10.1126/science.273.5275.595 8662544

[B15] Murzin AG Brenner SE Hubbard T Chothia C Scop - a Structural Classification of Proteins Database for the Investigation of Sequences and Structures Journal of Molecular Biology 1995 247 4 536 540 7723011 10.1006/jmbi.1995.0159

[B16] Orengo CA Michie AD Jones S Jones DT Swindells MB Thornton JM CATH - a hierarchic classification of protein domain structures Structure 1997 5 8 1093 1108 10.1016/S0969-2126(97)00260-8 9309224

[B17] Andreeva A Murzin AG Evolution of protein fold in the presence of functional constraints Current Opinion in Structural Biology 2006 16 3 399 408 10.1016/j.sbi.2006.04.003 16650981

[B18] Binkowski TA Adamian L Liang J Inferring functional relationships of proteins from local sequence and spatial surface patterns Journal of Molecular Biology 2003 332 2 505 526 10.1016/S0022-2836(03)00882-9 12948498

[B19] Kleywegt GJ Recognition of spatial motifs in protein structures Journal of Molecular Biology 1999 285 4 1887 1897 10.1006/jmbi.1998.2393 9917419

[B20] Laskowski RA Watson JD Thornton JM Protein function prediction using local 3D templates Journal of Molecular Biology 2005 351 3 614 626 10.1016/j.jmb.2005.05.067 16019027

[B21] Russell RB Detection of protein three-dimensional side-chain patterns: New examples of convergent evolution Journal of Molecular Biology 1998 279 5 1211 1227 10.1006/jmbi.1998.1844 9642096

[B22] Schmitt S Kuhn D Klebe G A new method to detect related function among proteins independent of sequence and fold homology Journal of Molecular Biology 2002 323 2 387 406 10.1016/S0022-2836(02)00811-2 12381328

[B23] Shulman-Peleg A Nussinov R Wolfson HJ Recognition of functional sites in protein structures Journal of Molecular Biology 2004 339 3 607 633 10.1016/j.jmb.2004.04.012 15147845 PMC7126412

[B24] Wallace AC Borkakoti N Thornton JM TESS: A geometric hashing algorithm for deriving 3D coordinate templates for searching structural databases. Application to enzyme active sites Protein Science 1997 6 11 2308 2323 10.1002/pro.5560061104 9385633 PMC2143595

[B25] Hamelryck T Efficient identification of side-chain patterns using a multidimensional index tree Proteins-Structure Function and Bioinformatics 2003 51 1 96 108 10.1002/prot.10338 12596267

[B26] Ashburner M Ball CA Blake JA Botstein D Butler H Cherry JM Davis AP Dolinski K Dwight SS Eppig JT Gene Ontology: tool for the unification of biology Nature Genetics 2000 25 1 25 29 10.1038/75556 10802651 PMC3037419

[B27] Willett P Barnard JM Downs GM Chemical similarity searching Journal of Chemical Information and Computer Sciences 1998 38 6 983 996

[B28] Berman HM Westbrook J Feng Z Gilliland G Bhat TN Weissig H Shindyalov IN Bourne PE The Protein Data Bank Nucleic Acids Research 2000 28 1 235 242 10.1093/nar/28.1.235 10592235 PMC102472

[B29] Brady GP Stouten PFW Fast prediction and visualization of protein binding pockets with PASS Journal of Computer-Aided Molecular Design 2000 14 4 383 401 10.1023/A:1008124202956 10815774

[B30] Pearson WR Empirical statistical estimates for sequence similarity searches Journal of Molecular Biology 1998 276 1 71 84 10.1006/jmbi.1997.1525 9514730

[B31] Gutteridge A Thornton JM Understanding nature's catalytic toolkit Trends in Biochemical Sciences 2005 30 11 622 629 10.1016/j.tibs.2005.09.006 16214343

[B32] Fawcett T An introduction to ROC analysis Pattern Recognition Letters 2006 27 8 861 874 10.1016/j.patrec.2005.10.010

[B33] Gold ND Jackson RM SitesBase: a database for structure-based protein-ligand binding site comparisons Nucleic Acids Res 2006 34 Database D231 234 10.1093/nar/gkj062 16381853 PMC1347425

[B34] Blaszczyk J Li Y Shi G Yan H Ji X Dynamic roles of arginine residues 82 and 92 of Escherichia coli 6-hydroxymethyl-7,8-dihydropterin pyrophosphokinase: crystallographic studies Biochemistry 2003 42 6 1573 1580 10.1021/bi0267994 12578370

[B35] Yeturu K Chandra N PocketMatch: a new algorithm to compare binding sites in protein structures BMC Bioinformatics 2008 9 543 10.1186/1471-2105-9-543 19091072 PMC2639437

[B36] Jmol: an open-source Java viewer for chemical structures in 3D http://www.jmol.org/

[B37] Laskowski RA Luscombe NM Swindells MB Thornton JM Protein clefts in molecular recognition and function Protein Science 1996 5 12 2438 2452 8976552 10.1002/pro.5560051206 PMC2143314

[B38] Xie L Bourne PE Detecting evolutionary relationships across existing fold space, using sequence order-independent profile-profile alignments Proceedings of the National Academy of Sciences of the United States of America 2008 105 14 5441 5446 10.1073/pnas.0704422105 18385384 PMC2291117

[B39] Liang MP Banatao DR Klein TE Brutlag DL Altman RB WebFEATURE: an interactive web tool for identifying and visualizing functional sites on macromolecular structures Nucleic Acids Research 2003 31 13 3324 3327 10.1093/nar/gkg553 12824318 PMC168960

[B40] Brylinski M Skolnick J A threading-based method (FINDSITE) for ligand-binding site prediction and functional annotation Proceedings of the National Academy of Sciences of the United States of America 2008 105 1 129 134 10.1073/pnas.0707684105 18165317 PMC2224172

[B41] Jiang XY Nariai N Steffen M Kasif S Kolaczyk ED Integration of relational and hierarchical network information for protein function prediction Bmc Bioinformatics 2008 9 350 364 10.1186/1471-2105-9-350 18721473 PMC2535605

[B42] Pal D Eisenberg D Inference of protein function from protein structure Structure 2005 13 1 121 130 10.1016/j.str.2004.10.015 15642267

