# Supplementary material for: BSSF: a fingerprint based ultrafast binding site similarity search and function analysis server
Source: BMC Bioinformatics. 2010 Jan 25;11:47. doi: 10.1186/1471-2105-11-47 (PMC3098077; doi:10.1186/1471-2105-11-47)

**Supporting materials**

**Table S1 Fragment type used in geometric hashing method.**

| Res_name | type | Fragment  Atom number | atoms |  |  |  |  |  |  |  |  |  |
| --- | --- | --- | --- | --- | --- | --- | --- | --- | --- | --- | --- | --- |
| ALA | 5 | 1 | CB |  |  |  |  |  |  |  |  |  |
| ARG | 6 | 4 | NE | CZ | NH1 | NH2 |  |  |  |  |  |  |
| ASN | 1 | 3 | CG | OD1 | ND2 |  |  |  |  |  |  |  |
| ASP | 7 | 3 | CG | OD1 | OD2 |  |  |  |  |  |  |  |
| CYS | 5 | 2 | CB | SG |  |  |  |  |  |  |  |  |
| GLN | 1 | 3 | CD | OE1 | NE2 |  |  |  |  |  |  |  |
| GLU | 7 | 3 | CD | OE1 | OE2 |  |  |  |  |  |  |  |
| HIS | 1 | 5 | CG | ND1 | CD2 | CE1 | NE2 |  |  |  |  |  |
| ILE | 5 | 3 | CG1 | CG2 | CD1 |  |  |  |  |  |  |  |
| LEU | 5 | 3 | CG | CD1 | CD2 |  |  |  |  |  |  |  |
| LYS | 6 | 2 | CE | NZ |  |  |  |  |  |  |  |  |
| MET | 5 | 3 | CG | SD | CE |  |  |  |  |  |  |  |
| PHE | 4 | 6 | CG | CD1 | CD2 | CE1 | CE2 | CZ |  |  |  |  |
| SER | 1 | 2 | CB | OG |  |  |  |  |  |  |  |  |
| THR | 1 | 2 | OG1 | CG2 |  |  |  |  |  |  |  |  |
| TRP | 4 | 9 | CG | CD1 | CD2 | NE1 | CE2 | CE3 | CZ2 | CZ3 | CH2 |  |
| TYR | 1 | 1 | OH |  |  |  |  |  |  |  |  |  |
| TYR | 4 | 6 | CB | CG | CD1 | CD2 | CE1 | CE2 | CZ |  |  |  |
| VAL | 5 | 3 | CB | CG1 | CG2 |  |  |  |  |  |  |  |

**Table S2 Pharmacophore types for residue fragments in fingerprint calculation.**

| Res_name | type | Fragment  Atom number | atoms |  |  |  |  |  |  |  |  |
| --- | --- | --- | --- | --- | --- | --- | --- | --- | --- | --- | --- |
| ALA | 2 | 1 | O |  |  |  |  |  |  |  |  |
| ALA | 3 | 1 | N |  |  |  |  |  |  |  |  |
| ALA | 5 | 1 | CB |  |  |  |  |  |  |  |  |
| ARG | 2 | 1 | O |  |  |  |  |  |  |  |  |
| ARG | 3 | 1 | N |  |  |  |  |  |  |  |  |
| ARG | 6 | 4 | NE | CZ | NH1 | NH2 |  |  |  |  |  |
| ASN | 1 | 3 | CG | OD1 | ND2 |  |  |  |  |  |  |
| ASN | 2 | 1 | O |  |  |  |  |  |  |  |  |
| ASN | 3 | 1 | N |  |  |  |  |  |  |  |  |
| ASP | 7 | 3 | CG | OD1 | OD2 |  |  |  |  |  |  |
| ASP | 2 | 1 | O |  |  |  |  |  |  |  |  |
| ASP | 3 | 1 | N |  |  |  |  |  |  |  |  |
| CYS | 2 | 1 | O |  |  |  |  |  |  |  |  |
| CYS | 3 | 1 | N |  |  |  |  |  |  |  |  |
| CYS | 5 | 2 | CB | SG |  |  |  |  |  |  |  |
| GLN | 1 | 3 | CD | OE1 | NE2 |  |  |  |  |  |  |
| GLN | 2 | 1 | O |  |  |  |  |  |  |  |  |
| GLN | 3 | 1 | N |  |  |  |  |  |  |  |  |
| GLU | 2 | 1 | O |  |  |  |  |  |  |  |  |
| GLU | 7 | 3 | CD | OE1 | OE2 |  |  |  |  |  |  |
| GLU | 3 | 1 | N |  |  |  |  |  |  |  |  |
| GLY | 2 | 1 | O |  |  |  |  |  |  |  |  |
| GLY | 3 | 1 | N |  |  |  |  |  |  |  |  |
| HIS | 1 | 5 | CG | ND1 | CD2 | CE1 | NE2 |  |  |  |  |
| HIS | 2 | 1 | O |  |  |  |  |  |  |  |  |
| HIS | 3 | 1 | N |  |  |  |  |  |  |  |  |
| ILE | 2 | 1 | O |  |  |  |  |  |  |  |  |
| ILE | 3 | 1 | N |  |  |  |  |  |  |  |  |
| ILE | 5 | 3 | CG1 | CG2 | CD1 |  |  |  |  |  |  |
| LEU | 2 | 1 | O |  |  |  |  |  |  |  |  |
| LEU | 3 | 1 | N |  |  |  |  |  |  |  |  |
| LEU | 5 | 3 | CG | CD1 | CD2 |  |  |  |  |  |  |
| LYS | 2 | 1 | O |  |  |  |  |  |  |  |  |
| LYS | 3 | 1 | N |  |  |  |  |  |  |  |  |
| LYS | 6 | 2 | CE | NZ |  |  |  |  |  |  |  |
| MET | 2 | 1 | O |  |  |  |  |  |  |  |  |
| MET | 3 | 1 | N |  |  |  |  |  |  |  |  |
| MET | 5 | 3 | CG | SD | CE |  |  |  |  |  |  |
| PHE | 2 | 1 | O |  |  |  |  |  |  |  |  |
| PHE | 3 | 1 | N |  |  |  |  |  |  |  |  |
| PHE | 4 | 6 | CG | CD1 | CD2 | CE1 | CE2 | CZ |  |  |  |
| PRO | 2 | 1 | O |  |  |  |  |  |  |  |  |
| PRO | 3 | 1 | N |  |  |  |  |  |  |  |  |
| SER | 1 | 2 | CB | OG |  |  |  |  |  |  |  |
| SER | 2 | 1 | O |  |  |  |  |  |  |  |  |
| SER | 3 | 1 | N |  |  |  |  |  |  |  |  |
| THR | 1 | 2 | OG1 | CG2 |  |  |  |  |  |  |  |
| THR | 2 | 1 | O |  |  |  |  |  |  |  |  |
| THR | 3 | 1 | N |  |  |  |  |  |  |  |  |
| TRP | 2 | 1 | O |  |  |  |  |  |  |  |  |
| TRP | 3 | 1 | N |  |  |  |  |  |  |  |  |
| TRP | 4 | 9 | CG | CD1 | CD2 | NE1 | CE2 | CE3 | CZ2 | CZ3 | CH2 |
| TYR | 1 | 1 | OH |  |  |  |  |  |  |  |  |
| TYR | 2 | 1 | O |  |  |  |  |  |  |  |  |
| TYR | 3 | 1 | N |  |  |  |  |  |  |  |  |
| TYR | 4 | 6 | CB | CG | CD1 | CD2 | CE1 | CE2 | CZ |  |  |
| VAL | 2 | 1 | O |  |  |  |  |  |  |  |  |
| VAL | 3 | 1 | N |  |  |  |  |  |  |  |  |
| VAL | 5 | 3 | CB | CG1 | CG2 |  |  |  |  |  |  |

**Table S3. List of binding site properties.**

| **Binding site properties** | **Average** |
| --- | --- |
| Binding sites per chain | 2.37 |
| Fragment number per binding site | 88.06 |
| Residue number per binding site | 30.09 |
| Type 1 fragment number per binding site | 8.19 |
| Type 2 fragment number per binding site | 30.09 |
| Type 3 fragment number per binding site | 30.09 |
| Type 4 fragment number per binding site | 9.24 |
| Type 5 fragment number per binding site | 3.48 |
| Type 6 fragment number per binding site | 3.56 |
| Type 7 fragment number per binding site | 3.39 |

**Figure S1 Ligands contained in the binding sites of fingerprint Z-score method’s predicted simiar pair 1DJY, 1PCM.**


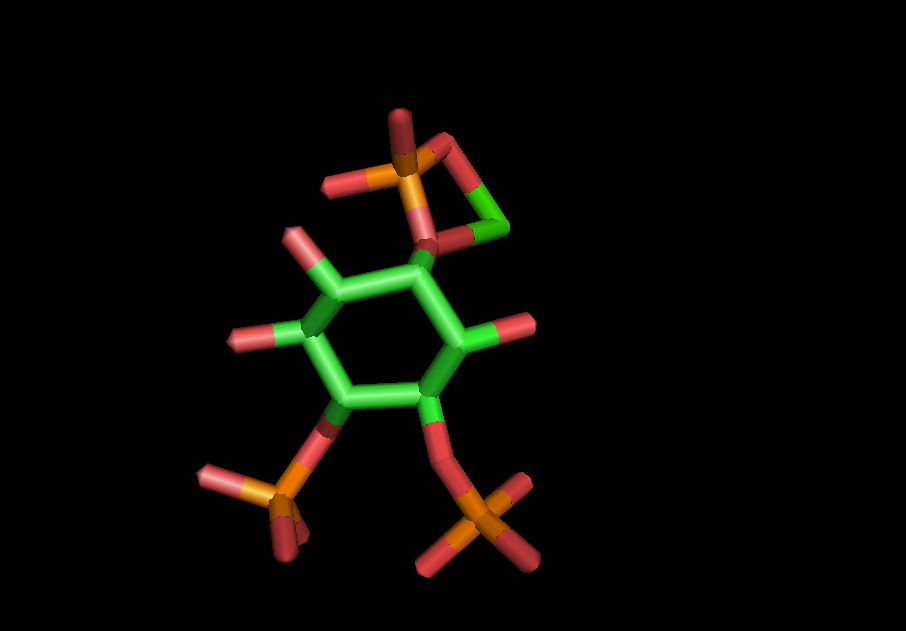


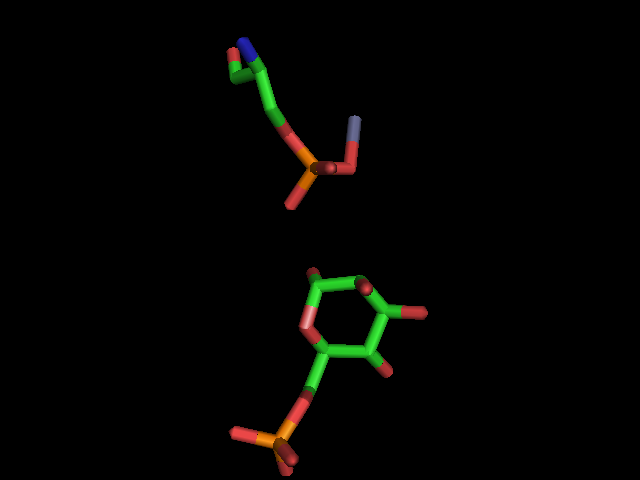

Supplement: Additional file 1 — Supporting material. It includes the fragment types in geometric hashing method and pharmacophore fingerprint. Also it contains the properties of binding sites. [file 1471-2105-11-47-S1.DOC]
